# *Elements* virtual rehabilitation improves motor, cognitive, and functional outcomes in adult stroke: evidence from a randomized controlled pilot study

**DOI:** 10.1186/s12984-019-0531-y

**Published:** 2019-05-15

**Authors:** Jeffrey M. Rogers, Jonathan Duckworth, Sandy Middleton, Bert Steenbergen, Peter H. Wilson

**Affiliations:** 1 The University of Sydney, Faculty of Health Sciences, Sydney, NSW Australia; 2 School of Design, RMIT, Melbourne, VIC Australia; 3 Nursing Research Institute, St Vincent’s Health Australia and Australian Catholic University, Sydney, NSW Australia; 4 Behavioural Science Institute, Radboud University, Nijmegen, The Netherlands; 5 Centre for Disability and Development Research (CeDDR) and School of Behavioural and Health Science, Australian Catholic University, Melbourne, VIC Australia

**Keywords:** Cognition, Motor activity, Rehabilitation, Stroke, Upper extremity, Virtual reality

## Abstract

**Background:**

Virtual reality technologies show potential as effective rehabilitation tools following neuro-trauma. In particular, the *Elements* system, involving customized surface computing and tangible interfaces, produces strong treatment effects for upper-limb and cognitive function following traumatic brain injury. The present study evaluated the efficacy of *Elements* as a virtual rehabilitation approach for stroke survivors.

**Methods:**

Twenty-one adults (42–94 years old) with sub-acute stroke were randomized to four weeks of *Elements* virtual rehabilitation (three weekly 30–40 min sessions) combined with treatment as usual (conventional occupational and physiotherapy) or to treatment as usual alone. Upper-limb skill (Box and Blocks Test), cognition (Montreal Cognitive Assessment and selected CogState subtests), and everyday participation (Neurobehavioral Functioning Inventory) were examined before and after inpatient training, and one-month later.

**Results:**

Effect sizes for the experimental group (*d* = 1.05–2.51) were larger compared with controls (*d* = 0.11–0.86), with *Elements* training showing statistically greater improvements in motor function of the most affected hand (*p* = 0.008), and general intellectual status and executive function (*p* ≤ 0.001). Proportional recovery was two- to three-fold greater than control participants, with superior transfer to everyday motor, cognitive, and communication behaviors. All gains were maintained at follow-up.

**Conclusion:**

A course of *Elements* virtual rehabilitation using goal-directed and exploratory upper-limb movement tasks facilitates both motor and cognitive recovery after stroke. The magnitude of training effects, maintenance of gains at follow-up, and generalization to daily activities provide compelling preliminary evidence of the power of virtual rehabilitation when applied in a targeted and principled manner.

**Trial registration:**

this pilot study was not registered.

## Introduction

Stroke is one of the most common forms of acquired brain injury (ABI), with around 60,000 new and recurrent strokes occurring every year in Australia alone [1]. The clinical outcome of stroke is variable but often includes persistent upper-limb motor deficits, including weakness, discoordination, and reduced speed and mobility [2], and cognitive impairments in information processing and executive function [3, 4]. Not surprisingly, stroke is a leading cause of disability worldwide, and the burden of stroke across all levels of the International Classification of Functioning (ICF) - body structures/function, activity, and participation - underlines the importance of interventions that can impact multiple domains of functioning [5, 6].

Recovery of functional performance following stroke remains a significant challenge for rehabilitation specialists [7, 8], but may be enhanced by innovation in the use of new technologies like virtual reality [9–12]. A critical goal is to find compelling ways of engaging individuals in their therapy by creating meaningful, stimulating and intensive forms of training [13]. The term, *virtual rehabilitation* (VR), is used to describe a form of training wherein patients interact with virtual or augmented environments, presented with the aid of technology [14, 15]. The technologies can be either commercial systems (e.g. Nintendo Wii, Xbox Kinect) or those customised specifically for rehabilitation. VR offers a number of advantages over traditional therapies, including the ability to engage individuals in the simulated practice of functional tasks at higher doses [16, 17], automated assessment of performance over time, flexibility in the scaling of task constraints, and a variety of reward structures to help maintain compliance [18].

While evaluation research is still in its infancy, recent systematic reviews and meta-analyses show that VR can enhance upper-limb motor outcomes in stroke [10, 11, 19], yielding treatment effects of medium-to-large magnitude [10, 11], and complementing conventional approaches to rehabilitation. VR has been shown to engender high levels of engagement in stroke patients undergoing physical therapy [20, 21] and training of even moderate intensity can afford functional benefits at the activity/skill level [9, 19]. In the specific case of upper-limb VR, however, there is little available evidence that these benefits transfer to *participation* [9]. Furthermore, most available data is on patients in chronic stages of recovery, with less on acute stroke [9]. Notwithstanding this, use of VR has begun to emerge in clinical practice, recommended in Australian and international stroke guidelines as a viable adjunct in therapy to improve motor and functional outcomes [22–24].

Until recently, most VR systems have been designed to improve motor functions, with cognitive outcomes often a secondary consideration in evaluation studies [9–11]. Notwithstanding this, treatments that target both motor and cognitive functions are indicated for stroke, given evidence that cognitive and motor systems overlap at a structural and functional level [25, 26], and work synergistically in a “perception-action cycle” [27] in stroke patients undergoing rehabilitation [28]. Recent studies provide preliminary evidence of improved attention and memory in stroke patients following motor-oriented VR [29–32], amounting to a small-to-medium effect on cognition [9]. When designed to address aspects of cognitive control and planning, VR has the potential to enhance dual-task control, resulting in better generalization of trained skills to daily functioning [33].

While evaluation research is still in its infancy, several recent customized systems (like *Elements*, the system evaluated here) have been deliberately designed to exploit factors known to enhance training intensity and motor learning. Informed by neuroscience and learning theory [for a recent review see 12], the *Elements* VR system was designed to enhance neuro-plastic recovery processes via: (1) an *enriched therapeutic environment* affording a natural form of user interaction via tangible computing and surface displays [34], which engage both the cognitive attention of participants and their motivation to explore training tasks; (2) concurrent *augmented feedback* (AF) on performance [35] offering participants additional information on the outcome of their actions to assist in re-building a sense of body position in space (aka *body schema*) and ability to predict/plan future actions; and (3) scaling of task challenges to the current level of motor and cognitive function [36], ensuring *dynamic scaffolding* of participants’ information processing and response capabilities. The *Elements* system, described in detail below and in earlier publications [37, 38], consists of a large (42 in.) tabletop surface display, tangible user interfaces, and software for presenting both goal-directed and exploratory virtual environments. Previous evaluations of the system in patients with traumatic brain injury showed improvements in both motor and cognitive performance, with transfer to activities of daily living [37, 39]. However, the impact of *Elements* in other forms of ABI, such as stroke, has not been evaluated.

The broad aim of current study was to evaluate the efficacy of the *Elements* VR interactive tabletop system for rehabilitation of motor and cognitive functions in sub-acute stroke, compared with *treatment as usual* (TAU). We were particularly interested in motor and cognitive outcomes, their relationship, and the transfer and maintenance of treatment effects. Training-related changes at the activity/skill level on standardized measures of motor and cognitive performance were investigated, together with functional changes. By offering an engaging, principled and customized form of interaction, we predicted that the *Elements* system would effect (i) greater changes on both motor and cognitive outcomes than with TAU alone; (ii) sustained benefits, as assessed over a short follow-up period, and (iii) transfer to everyday functional performance (i.e. participation).

## Methods

This study was approved by the relevant hospital and university Human Research Ethics Committees, and performed in accordance with their guidelines. As pilot research the study protocol was not registered.

### Participants

Stroke patients were recruited from the inpatient rehabilitation ward of a large tertiary hospital in Sydney, Australia. All patients had been admitted to the ward to address identified upper extremity dysfunction following a unilateral stroke, confirmed on neuroimaging. Additional inclusion criteria included: (1) ability to communicate in English, and understand and follow oral instructions; and (2) ability to maintain sitting balance unassisted. Exclusion criteria included: (1) a previous history of neurological (other than stroke), psychiatric, or developmental disorder; (2) loss of visual acuity preventing perception of visual material; or (3) under 18 years of age. Rehabilitation staff assisted in the identification of eligible candidates. All participants (or their carers) provided written informed consent prior to participation.

### Outcomes measures

The primary outcome was the *Box and Blocks Task* (BBT), which measures upper-limb motor skill [40]. The test is comprised of two hinged boxes (each 27 cm × 24 cm, with walls 8.5 cm high), separated by a vertical wooden barrier (15.2 cm high). One box is filled with 150 2.5-cm wooden cubes/blocks. The goal is to move as many blocks as possible over the barrier and into the opposite box in 60 s using one hand at a time. The BBT has been used frequently to assess motor function in patients with stroke, with demonstrated responsiveness to early post-stroke recovery [41] and high predictive validity, correlating in excess of 0.90 with more comprehensive examinations of upper limb motor function such as the Fugl-Meyer Assessment and the Action Research Arm Test [42], while offering the advantage of briefer administration.

Secondary outcomes inluded the *Montreal Cognitive Assessment* (MoCA), the *Groton Maze Learning Task* (GMLT) and the *Set Shift Task* (SST) from the CogState computerized assessment battery, and the *Neurobehavioural Functioning Inventory* (NFI). The MoCA is a brief (12-item) screening of general intellectual function across six domains: orientation, attention, language, visuospatial, memory, and executive function; scores below 26 out of 30 suggest cognitive impairment [43]. The MoCA has been repeatedly validated for assessing post-stroke cognitive status [44, 45], and its widespread use provides a standardized outcome with established clinical utility. At each assessment time point, a different one of three alternate forms was administered, with the order randomised and counter-balanced between participants.

The *Elements* tasks require attention, reasoning, and problem solving to discern task demands, and deduce the relationships and interactive principles at play (see descriptions below). Furthermore, *Elements* training requires participants to flexibly shift their response patterns from one task to another, or to inhibit an overlearned response in one particular task (Task 4: Go/No-Go). The GMLT and SST were therefore administered to measure these aspects of executive control [46]. The GMLT requires participants to deduce through trial-and-error learning a 28-step pathway hidden within a 10 × 10 grid. Participants repeat the same path several times, with the expectation subsequent trials will be completed more efficiently. In the SST participants are required to sort playing cards based on underlying rule sets related to either the color (red or black) or number shown on the card. The examinee must learn test rules through trial-and-error strategies, and flexibly shift sets as the test rules change over time. For both tasks, the outcome of interest was total errors, with lower scores indicative of better performance. Tasks from the CogState Battery were specifically designed to minimize practice effects and to be employed in repeated measures designs [46, 47].

Finally, the NFI is a 76-item patient reported outcome measure (PROM) of functional behaviour and symptoms in everyday life after ABI [48]. A range of symptoms commonly associated with neurological injury are distributed over six subscales: Depression, Somatic, Memory/Attention (aka Cognition), Communication, Aggression and Motor. The Motor sub-scale is not limited to upper-limb performance, but relates to whole-body strength, coordination and mobility, and the ability to complete daily tasks. The Cognition and Communication sub-scales measure the extent to which people lose track of time, forget important information, and have difficulty expressing themselves or comprehending others. The Somatic sub-scale measures physiological symptoms including headaches, nausea, dizziness and fatigue, while the Depression and Aggression sub-scales address emotional and behavioral issues, respectively. NFI total scores range from 70 to 350, with higher values indicating worsening ability to interact purposely with the environment [49].

### Procedure

Patients were stratified by age and type of stroke (ischemic or hemorrhagic), and then randomly allocated to the experimental (VR + TAU) or control (TAU alone) group. Concealed block randomization (1:1) was completed by breaking sequentially numbered opaque envelopes, pre-prepared by the study coordinator (JMR) using a random number generator (https://www.sealedenvelope.com/simple-randomiser/v1/lists). The research team and patients participating in this study were not blinded to assignment. Through medical chart review and patient interview, baseline information on sociodemographic and medical history, and current neurological and radiological data were collected. Assessment of motor, cognitive, and functional outcomes occurred at three time points: prior to *Elements* training (pre-test); immediately following training (post-test); and, one-month after the completion of training (follow-up).

The experimental and control group both received 3 h of daily conventional occupational and physiotherapy (i.e. TAU), provided by the treating allied health rehabilitation service at the hospital. TAU was individualized on the basis of collaborative care planning goals set by the patient and the treating team. Typical goals focused on range of motion exercises, muscle strengthening and coordination, and re-training of daily living skills (e.g. eating, grooming, toileting, dressing, transfers).

In addition to TAU, participants in the experimental group also received 12 sessions of VR, evenly distributed over four weeks. *Elements* training has previously been described in detail [37, 39], but briefly consisted of 30–40 min one-on-one sessions administered using a client-centered approach, with the level of individual task difficulty varied according to the participant’s level of performance and progress. Sessions were conducted with a registered psychologist, with training in virtual rehabilitation provided by the senior author, in a private therapy room at the hospital, free of distractions. Using four hand-held objects (i.e., the four “elements” in the shape of a circle, pentagon, triangle, and rectangle), the participant engaged with a virtual environment presented on a 42 in. touchscreen LCD panel (Multitaction™) with inbuilt CPU. *Elements* tasks included: Task 1 (Bases) consists of the home base and four potential movement targets, all 78 mm in diameter. The circular targets are cued in a fixed order (east, north, west, south) using an illuminated border. Task 2 (Random Bases) has the same configuration of targets, but they are highlighted in random order. Task 3 (Chase Task) begins with a blank screen. A target circle then appears randomly in one of nine locations. These locations are configured along three radials emanating from the home base. Task 4 (Go/No-Go) uses the same target positions as Task 3, however, additional distractor targets (a pentagon, triangle and rectangle) appear. Participants are instructed to place the object on the circular targets only and to resist moving to distractors. Tasks 5, 6 and 7 require participants to explore the virtual environment, by creating various shapes and sounds through movement.

**Fig. 1:**
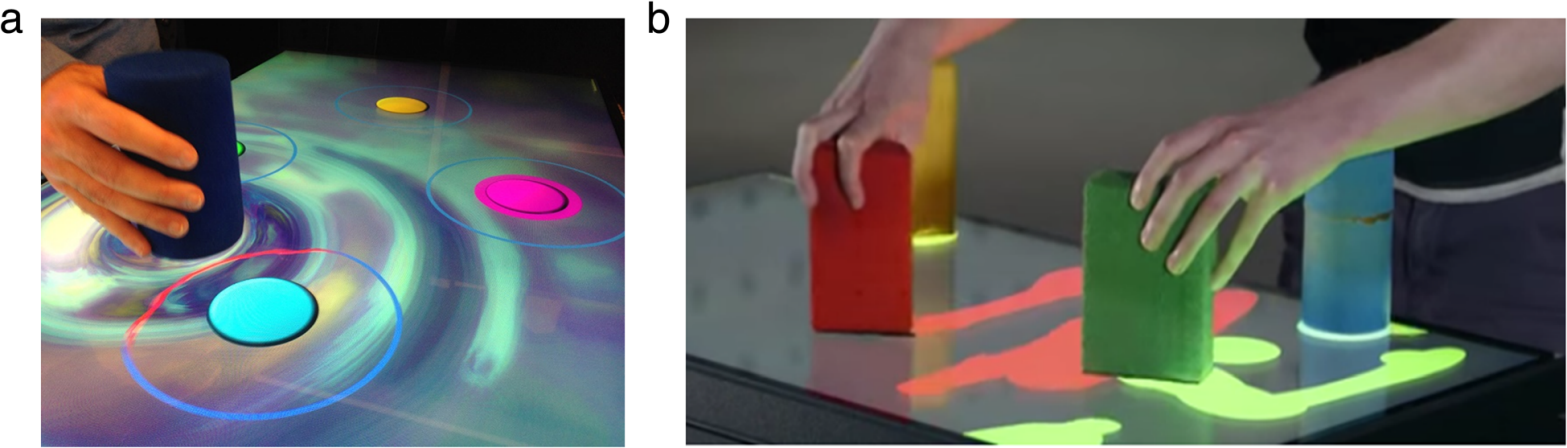
Examples of the *Elements* (**a**) goal-directed Bases task with visual augmented feedback, and (**b**) exploratory Squiggles task

During each session, participants progressed through a series of unimanual, goal-directed tasks (Tasks 1–4), followed by an exploratory task (Tasks 5–7) of their choosing (Fig. 1). The four goal-directed tasks involve movement (lift or slide using one hand) and placement of the circular hand-held object toward select targets; performance metrics (speed and accuracy) for the least and most affected hand are logged at the end of each task, and plots showing progress over time are discussed at the end of each session (i.e. explicit feedback). During the tasks, augmented auditory and visual feedback is presented in real-time, reinforcing movement-related attributes like speed, trajectory and endpoint contact (i.e., implicit feedback). For example, visual AF includes a fading trail of a hand-held object’s path, a waxing luminescence around targets as an object approaches, and ripple effects when an object is placed on a target. Auditory AF includes one tonal source that increases in pitch with greater movement speed, a second tone that increases in pitch as an object approaches a target, and a third tone emitted when an object is placed on a target.

The three exploratory tasks included Mixer (Task 5), Squiggles (Task 6), and Swarm (Task 7). Mixer consists of nine circles in a 3 × 3 grid. Moving the circular hand-held object close to a circle starts to activate its sound and spinning border animation. The pitch and tone of the sound vary according to the hand-held object’s proximity to the circle. Participants can activate different combinations of circles at any time to produce an overall soundscape. Squiggles presents a blank display upon which participants can draw lines and shapes by sliding any of the four different hand-held objects across the screen. As each object is moved, a trail animation is drawn along its path and a musical tone plays. Once the participant lifts the object, the trail animates and moves across the screen. Each object has a unique visual trail and musical tone. Swarm encourages bimanual control to explore the audiovisual relationships between all four hand-held objects. When placed on the screen, multiple colored shapes slowly gravitate toward and swarm around the base of each hand-held object. As each object is moved, its associated swarm follows. The movement, color, size and sound characteristics of each swarm change when the distance between objects is altered and the different swarms overlap/interact.

The *Elements* tasks were designed to exploit a range of factors known to enhance training intensity and motor learning: concurrent AF [50]; embodied interaction using tangible interfaces [51, 52], and heightened task engagement using a combination of goal-directed and exploratory tasks. These factors are thought to enhance not only the motor control/learning processes that underpin movement skills [53], but also aspects of cognitive control which, together, improve the transfer of skill to everyday behavior [14, 54]. The principled design in VR is important; customized systems like *Elements* tend to be more effective than off-the-shelf commercial gaming systems [9, 12], which encourage nonspecific movements and may not permit participant-specific settings.

### Data analysis

With a desired power of 0.80, and the expectation of a *medium* treatment effect (Cohen’s *d* > 0.50) based on reported changes in motor activity (i.e. Box and Blocks test) in comparable VR studies [31, 55, 56], a sample size of 10–12 participants per group was determined as adequate (G*Power 3.1.7 program, http://www.gpower.hhu.de/). This calculation was also qualitatively consistent with the sample sizes in methodologically similar proof of concept studies of VR for stroke [57–59].

All data was checked for normality using Shapiro-Wilk’s and Levene’s tests; where violations were detected the non-parametric alternative Mann-Whitney U test was applied. The analysis of training effects was conducted in three parts. First, scores on each outcome measure at each time point (pre-test, post-test, follow-up) were compared between the two groups using a series of 95% CI’s and independent *t*-tests. To control the rate of false positives in the planned multiple comparisons, the Benjamini–Hochberg procedure [60], with a false discovery rate of 0.07, was applied to determine the number of significant results in each family of tests (6 motor outcomes, 6 cognitive outcomes, 12 participation outcomes). Second, for each treatment group, the significance of pre-test to post-test and pre-test to follow-up change on each outcome measure was analyzed using dependent *t*-tests, and the magnitude of each effect reported as Cohen’s *d*. Effect sizes were interpreted according to the conventions of Cohen [61]: *small* ≥ 0.2; *medium* ≥ 0.5; and, *large* ≥ 0.8. Third, pre-post change scores were applied to calculate the *Proportion of Achieved Recovery* for each group. Calculated as [100 x (score_post-test_ – score_pre-test_) / (score_maximum_ – score_pre-test_)], proportional recovery normalizes change scores across the severity spectrum for comparing recovery among cohorts with both severe initial impairment (and hence greater room for improvement) and more mild initial impairment [62]. Proportional Recovery Scores were not used to predict recovery. For the MoCA (higher scores denote better outcomes) and the NFI (lower scores denote better outcomes), the maximum scores were 30 and 70, respectively. For the BBT, gender-, hand dominance-, and age-based healthy adult normative data published by the original authors were fitted to each individual participant and used to define the maximum score [63].

## Results

A total of 21 patients were recruited to the study between March 2016 and September 2017, including 10 patients randomized to the experimental group, and 11 patients randomized to the control group. All enrolled participants completed the study, and there were no dropouts or adverse events. The demographic and clinical characteristic of the participants in both groups are shown in Table 1. There were no statistically significant between-group differences in age, gender, education, stroke location and severity, or time since stroke.

**Table 1:** Demographic, neurological, and functional characteristics of the experimental and control group at baseline

	Virtual Rehabilitation (*n* = 10)	Treatment As Usual (*n* = 11)	Comparison Test
Age (years)^a^	64.3 (17.4), 42–94 (66)	64.6 (12.0), 52–79 (69)	*t* = − 0.05, *p* = 0.96
Gender^b^			χ^2^ = 0.6, *p* = 0.80
Male	4 (40)	5 (45)	
Female	6 (60)	6 (55)	
Education (years)^a^	13.5 (2.1), 10–16 (14)	12.5 (1.9), 10–15 (12)	*t* = 1.20, *p* = 0.24
Rehab NIHSS^a^	3.0 (1.8), 0–5 (3.5)	2.3 (1.6), 0–4 (2.5)	*t* = 0.67, *p* = 0.52
Time since stroke (days)^a^	22.8 (14.8), 8–44 (24)	30.0 (15.9), 10–62 (32)	*t* = − 1.07, *p* = 0.30
Ischemic Stroke^b^	9 (90)	9 (82)	
Hemorrhagic Stroke^b^	1 (10)	2 (18)	
Left-sided lesion^b^	4 (40)	5 (45)	
Right-sided lesion^b^	6 (60)	6 (55)	
Oxfordshire Classification			χ^2^ = 4.96, *p* = 0.17
TACI/H^b^	5 (50)	1 (9)	
LACI/H^b^	1 (10)	2 (18)	
PACI/H^b^	2 (20)	6 (55)	
POCI/H^b^	2 (20)	2 (18)	
MoCA baseline^a^	18.4 (2.5), 14–22 (18.5)	19.2 (4.1), 12–24 (18)	*t* = −0.53, *p* = 0.61
BBT baseline, MAH^a^	21.8 (12.8), 12–43 (15)	21.5 (8.1), 13–34 (20)	*t* = 0.08, *p* = 0.94
BBT baseline, LAH^a^	45.1 (7.9), 30–56 (44.5)	44.5 (8.3), 30–55 (45)	*t* = 0.18, *p* = 0.86
GMLT Errors baseline	107.8 (12.3), 92–124 (104)	110.6 (14.7), 92–125 (117)	*t* = − 0.46, *p* = 0.65
Set Shift Errors baseline	64.9 (10.1), 50–79 (68.5)	64.6 (12.7), 47–89 (65)	*t* = 0.07, *p* = 0.95
NFI baseline	181.1 (36.6), 124–255 (180.5)	182.8 (43.1), 97–227 (189)	*t* = − 0.10, *p* = 0.92

^a^Mean (SD) range (median); ^b^No (%). Note: *GMLT* CogState Groton Maze Learning Task, *LACI/H* lacunar infarct/ hemorrhage, *LAH* Less Affected Hand, *MAH* Most Affected Hand, *MoCA* Montreal Cognitive Assessment, *NFI* Neurobehavioral Functioning Inventory, *NIHSS* National Institute of Health Stroke Scale range 0-24; *PACI/H* partial anterior circulation infarct/ hemorrhage, *POCI/H* posterior circulation infarct/ hemorrhage, *TACI/H* total anterior circulation infarct/ hemorrhage

Baseline (or pre-test) motor (BBT), cognitive (MoCA and CogState tasks), and functional (NFI) status were equivalent between groups (Tables 1-3). Both groups showed significant training-related improvement on motor and cognitive functions (Table 2), and functional status (Table 3). However, the magnitude of effect sizes were substantially larger for the intervention group (Cohen’s *d* = 1.05–2.51) compared with the control group (Cohen’s *d* = 0.11–0.86), as shown in Tables 2 and 3. In particular, on pre-post difference scores, the intervention group showed significantly greater improvement in motor function (BBT) in the hand most affected by their stroke (*p* = 0.008), and on all measures of cognitive function (MoCA and CogState tasks; *p* ≤ 0.001), compared with the control group. Group differences were also evident on (self-report) measures of functional performance: NFI motor (*p* ≤ 0.001), NFI cognitive (*p* = 0.022), and NFI communication (*p* = 0.002) function.

**Table 2:** Motor and cognitive outcomes for the experimental and control groups at pre-test, post-test and one-month follow-up

	VR + TAU					TAU					Group effect on pre-post difference score (*t* or *U*, *p*)^c^	Group effect on pre-test to follow-up difference score (*t* or *U*, *p*)^c^
Primary Outcome^a^	Pre-test	Post-test	Follow-up	Pre-post difference score^b^	Effect size *d*^b^	Pre-test	Post-test	Follow-up	Pre-post difference score^b^	Effect size *d*^b^		
Motor												
BBT-MAH	21.8 (12.8)	39.1 (14.5)	40.8 (15.5)	17.3 (8.6), *p* < 0.001	1.3	21.5 (8.1)	29.8 (11.2)	30.9 (11.5)	8.4 (5.3), *p* < 0.001	0.9	21.50, 0.008*	23.00, 0.011*
BBT-LAH	45.1 (7.9)	57.6 (5.4)	60.6 (5.6)	12.5 (7.7), *p* = 0.001	1.9	44.5 (8.3)	50.9 (8.2)	50.9 (7.8)	6.5 (5.7), *p* = 0.004	0.8	2.05, 0.054	−3.44, 0.003*
BBT Total	66.9 (14.1)	96.7 (12.6)	101.4 (14.7)	29.8 (11.5), *p* < 0.001	2.2	65.9 (10.3)	80.7 (11.9)	81.8 (11.4)	14.8 (7.8), *p* < 0.001	1.3	3.52, 0.002*	9.50, < 0.001*
Cognitive												
MoCA	18.4 (2.50)	24.8 (2.6)	25.4 (2.6)	6.40 (1.3), *p* < 0.001	2.5	19.2 (4.0)	21.4 (3.6)	21.8 (3.5)	2.2 (0.9), *p* = 0.004	0.6	0.50, < 0.001*	− 7.41, < 0.001*
GMLT Errors	107.8 (12.3)	87.5 (9.5)	76.1 (10.5)	20.3 (8.4), *p* < 0.001	1.9	110.6 (14.7)	108.0 (13.2)	101.1 (14.5)	2.5 (6.1), *p* = 0.198	0.2	−5.57, < 0.001*	4.32, < 0.001*
Set Shift Errors	64.9 (10.1)	46.9 (6.2)	33.3 (7.0)	18.0 (10.3), *p* < 0.001	2.2	64.6 (12.7)	61.0 (11.5)	58.9 (12.6)	3.5 (6.1), *p* = 0.082	0.3	−3.95, 0.001*	7.43, < 0.001*

^a^Mean (*SD*); ^b^within-group dependent t-test comparison of pre-test vs. post-test; ^c^between-group independent t-test comparison of VR+TAU vs. TAU; * *p* < Benjamini-Hochberg critical value. *BBT* Box and Blocks Test, *GMLT* CogState Groton Maze Learning Task, *LAH* Less Affected Hand, *MAH* Most Affected Hand, *MoCA* Montreal Cognitive Assessment, *TAU* Treatment as Usual, *VR* Virtual Rehabilitation

**Table 3:** Functional outcomes for the experimental and control groups at pre-test, post-test and one-month follow-up

	VR + TAU					TAU					Group effect on pre-post difference score (*t* or *U*, *p*)^c^	Group effect on pre-test to follow-up difference score (*t* or *U*, *p*)^c^
NFI Sub-scale^a^	Pre-test	Post-test	Follow-up	Pre-post difference score^b^	Effect size *d*^b^	Pre-test	Post- test	Follow-up	Pre-post difference score^b^	Effect size *d*^b^		
Motor	32.2 (3.9)	24.4 (4.0)	21.5 (2.9)	7.8 (1.7), *p* < 0.001	2.0	30.6 (4.1)	27.5 (4.2)	26.7 (5.2)	3.1 (1.8),*p* < 0.001	0.8	6.00, < 0.001*	5.85, < 0.001*
Cognition	56.9 (18.1)	39.8 (14.2)	34.6 (12.6)	17.1 (7.6), *p* < 0.001	1.1	51.8 (19.2)	42.4 (14.9)	39.3 (13.7)	9.5 (6.5),*p* = 0.001	0.6	−2.50, 0.022*	2.33, 0.031*
Depression	32.6 (9.1)	24.5 (6.6)	22.1 (5.8)	8.1 (4.4), *p* < 0.001	1.0	33.9 (13.9)	30.6 (10.6)	28.2 (9.7)	3.4 (6.8),*p* = 0.130	0.3	−1.89, 0.075	1.82, 0.085
Somatic	20.4 (6.3)	16.4 (5.5)	16.2 (4.4)	4.0 (2.1),*p* < 0.001	0.7	19.7 (4.1)	17.3 (2.9)	18.3 (4.0)	2.5 (3.1),*p* = 0.025	0.7	−1.33, 0.200	2.02, 0.058
Commun	34.9 (6.5)	25.9 (6.9)	22.5 (5.3)	9.0 (4.0),*p* < 0.001	1.3	31.8 (12.0)	30.6 (10.6)	28.3 (9.8)	1.3 (5.6),*p* = 0.467	0.1	−3.62, 0.002*	4.62, < 0.001*
Aggression	15.8 (4.7)	14.6 (5.7)	13.9 (4.9)	1.2 (2.3), *p* = 0.612	0.2	14.6 (4.4)	14.2 (4.1)	13.6 (5.2)	0.4 (1.8), *p* = 0.831	0.1	0.89,0.383	0.98, 0.347

^a^Mean (*SD*); ^b^within-group dependent t-test comparison of pre-test vs. post-test; ^c^between-group independent t-test comparison of VR+TAU vs. TAU;. * *p* < Benjamini-Hochberg critical value. Commun: Communication; *NFI* Neurobehavioral Functioning Inventory, *TAU* Treatment as Usual, *VR* Virtual Rehabilitation

The gains (37.4–57.2% c.f. 20.5–21.5%) made over the four week training period by the experimental group equated to a proportional recovery roughly 2–3 times that of the control group (Table 4). Furthermore, improvements shown by the experimental group as a function of training were maintained at the one-month follow-up assessment (Tables 2-3): on difference scores between pre-test and follow-up, the intervention group performed significantly better than controls on motor (*p* ≤ 0.01), cognitive (*p* < 0.001), and functional outcomes (*p* ≤ 0.03).

**Table 4:** Proportion of Achieved Recovery from pre-test to post-test for the experimental and control groups

	Virtual Rehabilitation (*n* = 10)	Treatment As Usual (*n* = 11)	Comparison Test^b^
BBT Total^a^	41.5% (15.7%), 19–75%	20.5% (11.7%), 8–48%	*t* = 3.49, *p* = 0.002**
MoCA^a^	57.2% (15.0%), 29–88%	20.7% (5.7%), 11–29%	*t* = 7.49, *p* < 0.001***
NFI Total^a^	37.4% (13.0%), 23–62%	21.5% (16.5%), 0–39%	*t* = 2.45, *p* = 0.02*

^a^Mean (SD) range; ^b^between-group independent t-test comparison of VR+TAU vs. TAU. Note: * *p* < .05; ** *p* < .01; *** *p* < .001. *BBT* Box and Blocks Test, *NFI* Neurobehavioral Functioning Inventory, *MoCA* Montreal Cognitive Assessment, *TAU* Treatment as Usual, *VR* Virtual Rehabilitation

## Discussion

Persistent and disabling [64] upper-limb motor dysfunction is prevalent after stroke [65], and seriously undermines performance of daily activities. As well, cognitive impairment is a common [7, 66] and persistent [67] sequela of stroke, irrespective of stroke severity [68, 69] and a so-called “good” neurological recovery [66]. Neuroplasticity can be enhanced by training that engages the cognitive abilities of the learner, increases the motivation to succeed in the task [70], and provides multimodal (i.e. augmented) feedback that can be used as a training signal for skill development [71]. Virtual-reality based approaches afford these qualities, fostering active participation [72], motor learning, and rehabilitation efficacy [73, 74].

The current study evaluated the effects of a short course of *Elements* VR training for rehabilitation of adult stroke. Specifically, the *Elements* system was designed to promote the re-acquisition of upper-limb skill and cognitive-motor coupling via the use of concurrent AF, tangible interaction design, and game-like features that enhance user engagement. Training-related change was measured at multiple levels of the ICF [5, 8] including body structures/function (MoCA), activity/skill (BBT), and everyday participation (NFI). As predicted, *Elements* training was shown to be more effective than conventional treatment as usual, enhancing performance across measures of motor, cognitive, and everyday function. The treatment effects for *Elements* across measures are discussed in more detail below.

In the current study, treatment as usual with conventional occupational and physiotherapy was associated with a significant improvement in upper limb function as measured with the BBT (*d* = 0.78–0.86). However, as expected, participants also receiving *Elements* training experienced a greater improvement in performance on the BBT (*d* = 1.27–1.85). In terms of proportional recovery, gains for the experimental group were two- to three-fold greater than controls, and over the one-month follow-up period the improvements in motor function were maintained. We reiterate, however, that proportional recovery scores should not be used here to predict longer-term recovery.

As with the BBT motor outcomes, the improvement in cognitive status using the MoCA screening tool was both statistically and clinically significant. Specifically, participants in the experimental condition experienced greater improvements (*d* = 2.57) in general intellectual status compared to control participants (*d* = 0.51), reflecting a nearly three-fold increase in the proportion of recovery achieved. Furthermore, immediate gains in cognitive status made following *Elements* training were again retained at the one-month follow-up.

The cognitive assessment was supplemented with the GMLT and the SST from the CogState computerized battery, providing unique, non-overlapping information, with no significant correlations with MoCA outcomes at any of the three data collection time points (Table 5). Notably, the CogState tasks revealed improvements in aspects of executive functioning following *Elements* training (*d* = 1.86–2.15), that were maintained at follow-up, but were only minimally present in the control group participants receiving just treatment as usual (*d* = 0.19–0.30).

**Table 5:** Experimental group correlations (2-tailed) between cognitive outcomes at the pre-test, post-test, and one-month follow-up time-points. Shaded cells represent correlations within a time-point

	1	2	3	4	5	6	7	8	9
1. Pre-test MoCA	–								
2. Pre-test Cogstate GMLT Errors	−0.30	–							
3. Pre-test CogState Set Shift Errors	0.14	0.25	–						
4. Post-test MoCA	0.88^**^	− 0.32	0.34	–					
5. Post-test Cogstate GMLT Errors	0.21	0.73	0.62	0.23	–				
6. Post-test CogState Set Shift Errors	0.10	0.41	0.26	−0.08	0.39	–			
7. Follow-up MoCA	0.83^**^	−0.43	0.24	0.96^**^	0.11	−0.26	–		
8. Follow-up Cogstate GMLT Errors	0.02	0.39	0.37	0.12	0.54	0.41	0.12	–	
9. Follow-up CogState Set Shift Errors	0.09	0.09	0.49	0.22	0.35	0.47	0.17	0.30	–

Note: ** *p* < .01; *GMLT* Groton Maze Learning Task, *MoCA* Montreal Cognitive Assessment

In addition to improvement on these untrained cognitive and motor tasks, functioning in everyday life situations was also better improved in the experimental group (*d* = 1.05–1.97) compared to the control group (*d* = 0.11–0.75). In particular, self-rated performance of everyday motor, cognitive, and communication activities on the NFI all significantly improved following *Elements* training, with gains sustained at the one-month follow-up. The benefits of *Elements* VR appear to extend beyond trained upper-limb function, which provides encouraging preliminary evidence that use of the system can help promote engagement in a range of meaningful personal and social activities, and may address some of the chronic participation restrictions many stroke survivors experience [75]. The somatic sub-scale of the NFI showed no significant difference between the two groups. While somatic symptoms can be common in stroke patients [76, 77], *Elements* VR was not designed to address them. However, the lack of any significant group difference may also reflect the general safety and tolerability of the *Elements* systems in stroke patients. In contrast, virtual reality technologies utilizing head-mounted displays or immersive environments have been associated with adverse somatic “cybersickness” reactions in some acute neurological patients [20, 78].

Aggression sub-scale ratings were minimal in both groups at all three time points. The NFI was developed for traumatic brain injury populations, but neurobehavioral disturbances in this domain may be relatively rare after stroke [79]. Finally, stroke survivors often have mental health concerns [80], and virtual reality therapy can enhance stroke patients’ psychological well-being [25]. In the current study *Elements* training was associated with some improvement in mood symptoms, possibly by increasing motivation, and decreasing motor and cognitive deficits [81]. However, the between group difference failed to reach statistical significance (*p* = 0.075). Future studies of VR in stroke are encouraged to examine the potential impact of interventions on improving psychological well-being.

Overall, these results are consistent with previous reports that purpose-built virtual reality approaches improve upper limb function after stroke, as measured with the BBT e.g., [54, 58, 73, 82], or other common upper limb instruments such as the Fugl-Meyer Assessment e.g., [54, 57, 58, 82–85] or Action Research Arm Test [59, 84]. Gains in motor function in the current study exceeded those produced from treatment as usual, which has not always been the case when commercially available virtual reality systems are used for stroke e.g., [20, 31, 86].

Cognitive and functional outcomes have not typically been a focus of past research [87], and there is a need for VR approaches that address non-motor deficits [88]. While purpose-built VR approaches have struggled to consistently outperform treatment as usual in regard to cognitive [89, 90] and functional outcomes [54, 58, 83, 85, 86] after stroke, the current findings add to a small number of reports of improvement on attention and memory tasks [91] and daily activities on the Motor Activity Log [92]. Notably, the treatment as usual approach in the current study had limited impact on either of these important domains, highlighting previous observations that gains made in conventional stroke rehabilitation therapy are typically restricted to trained physical functions and motor activities [93, 94]. In addition, while preliminary, the effect size for MoCA improvements in the experimental group (*d* = 2.51) exceeded the previously reported pooled effect size (g = 0.29) of post-stroke cognitive remediation on general intellectual function, derived from outcomes on the MoCA and Mini Mental State Exam screening instruments [95]. As the neurological deficits arising after stroke span both the motor and cognitive domains, it is logical that rehabilitation programs that obtain maximum effectiveness will be those that can simultaneously address both domains [90]. Based on the promising initial results of the *Elements* system, future stroke rehabilitation research is encouraged to similarly adopt principled strategies for stroke rehabilitation that make use of grounded and embodied cognition theory [27, 96, 97] to merge cognitive and motor intensive training.

In sum, *Elements* training appeared effective at enhancing the impact of standard physical rehabilitation following stroke, inducing additional motor, cognitive, and functional gains. The capacity of the *Elements* system to produce meaningful and sustained improvement appears to be derived from the system’s capability to effectively leverage cognitive-motor interactions within a game-like format. The result was an engaging and motivating experience [73, 74], wherein the additional time in therapy was well received by a sub-acute neurological population of adults and older adults, and produced improvements in multiple domains of function that generalized to daily life behaviors.

### Limitations

We acknowledge the limitations of this research. First, although comparable with other proof of concept studies of VR for stroke [21, 57–59], the current study was based on a modest sample of 21 participants. While the effect sizes of the experimental group were all *large* [61], suggesting the study was adequately powered, replication in a bigger sample is encouraged, permitting analysis of potential moderators of the extent of proportional recovery achieved, and enhancing the generalizability of the findings. In particular, the current cohort was predominantly of *mild* severity (as per the NIHSS), and due to ischemic stroke. These characteristics are representative of the natural incidence of stroke, wherein > 75% of strokes suffered by older adults are ischemic [98], and more than half of all ischemic strokes are classified on the NIHSS as mild [99]. However, it is uncertain how the current findings apply to hemorrhagic stroke and patients with more severe baseline neurological deficits.

Active control group designs are typically preferred for their capability to presumably control for Hawthorne effects and other biases when comparison groups are not balanced in terms of time in therapy. While the design of the current study was informed by our recent meta-analysis of VR for stroke that suggested effect sizes are not over-estimated in studies that lack of an active control group [9], we acknowledge that the intervention group received around 7 h of additional activity over the course of the current study. Although this represented only a small increase (12%) above the approximate 60 h of conventional rehabilitation the control group received, future studies could attempt to minimize any potential confound by balancing the “dose” of training between groups. Furthermore, functional outcomes in the current study were derived from subjective self-report. While PROMs can be more predictive of rehabilitation outcomes than objective performance measures [100], they may be vulnerable to recall and expectancy bias, and post-stroke impairments in self-awareness [101]. Independent corroboration of functional outcomes from a reliable informant is recommended for future studies. Finally, outcomes assessors were not blinded, which can increase the risk of detection bias [102].

Follow-up in the current study occurred only one-month after the completion of training. However, rehabilitation gains realized in the first six-months after stroke may not be maintained [2], and longer follow-up periods are encouraged to provide more robust data on the durability of VR. In addition, the resources required to implement the *Elements* VR program were admittedly substantial, and a barrier to widespread adoption into routine clinical practice [103]. Development and evaluation of a lower-cost, portable, tablet-based version of the *Elements* system is therefore underway. Finally, while the validity of predicting recovery after stroke using the Proportion of Achieved Recovery formula has recently been challenged [104, 105], when used simply as a tool for comparing recovery we note only two patients in the experimental group, and none in the control group, achieved what could be described as a complete or near-complete recovery (e.g. achieved ≥70% of their proportional recovery [106]) during the four-week training period. To achieve the dose and intensity of therapy needed for neural reorganization and functional change, exploration of alternatives and adjuncts to clinician-led inpatient rehabilitation are encouraged, such as VR that can also be delivered in the community [107], and that patients can self-administer [108, 109].

## Conclusions

Results from the *Elements* VR system are one step toward the goal that “someday … people with stroke will unknowingly receive therapy while playing fun and challenging video games (pp. 440)” [110]. Applied concurrent to conventional occupational and physiotherapy, a course of VR using goal-directed and exploratory upper-limb movement tasks can facilitate greater recovery of both motor and cognitive function after stroke. The magnitude of training effects, maintenance of gains at follow-up, and generalization to daily activities, provide compelling preliminary evidence of the power of VR when applied in a targeted, and principled manner.

## Abbreviations

ABI: acquired brain injury
AF: augmented feedback
BBT: Box and Blocks Test
CI: Confidence Interval
GMLT: Groton Maze Learning Task
ICF: International Classification of Functioning
MoCA: Montreal Cognitive Assessment
NFI: Neurobehavioral Functioning Inventory
NIHSS: National Institutes of Health Stroke Scale
PROM: patient-reported outcome measure
SST: Set Shift Task
TAU: treatment as usual
VR: virtual rehabilitation
